# The Impact of PEO and PVP Additives on the Structure and Properties of Silk Fibroin Adsorption Layers

**DOI:** 10.3390/polym17131733

**Published:** 2025-06-21

**Authors:** Olga Yu. Milyaeva, Kseniya Yu. Rotanova, Anastasiya R. Rafikova, Reinhard Miller, Giuseppe Loglio, Boris A. Noskov

**Affiliations:** 1 Department of Colloid Chemistry, St. Petersburg State University, Universitetsky pr. 26, 198504 St. Petersburg, Russia; rotanova.ksyusha@gmail.com (K.Y.R.); nastya.rafikova.2000@mail.ru (A.R.R.); b.noskov@spbu.ru (B.A.N.); 2 Institute of Condensed Matter Physics, Technische Universität Darmstadt, D-64289 Darmstadt, Germany; reinhard.miller@pkm.tu-darmstadt.de; 3 Institute of Condensed Matter Chemistry and Technologies for Energy, 16149 Genoa, Italy; giuseppe.loglio@ge.icmate.cnr.it

**Keywords:** silk fibroin, air–water interface, polyethylene oxide, polyvinylpyrrolidone, adsorption layers, dilational surface visco-elasticity, surface ellipsometry, atomic force microscopy

## Abstract

Materials formed with a base of silk fibroin (SF) are successfully used in tissue engineering since their properties are similar to those of natural extracellular matrixes. Mixing SF with different polymers, for example, polyethylene oxide (PEO) and polyvinylpyrrolidone (PVP), allows the production of fibers, hydrogels, and films and their morphology to be controlled. The impact of PEO and PVP on formation and structure of SF adsorption layers was studied at different was studied at different polymer concentrations (from 0.002 to 0.5 mg/mL) and surface lifetimes. The protein concentration was fixed at 0.02 and 0.2 mg/mL. These concentrations are characterized by different types of spontaneously formed structures at the air–water interface. Since both synthetic polymers possess surface activity, they can penetrate the fibroin adsorption layer, leading to a decrease in the dynamic surface elasticity at almost constant surface tension and a decrease in ellipsometric angle Δ and adsorption layer thickness. As shown by AFM, the presence of polymers increases the porosity of the adsorption layer, due to the possible arrangement of protein and polymer molecules into separate domains, and can result in various morphology types such as fibers or tree-like ribbons. Therefore, polymers like PEO and PVP can be used to regulate the SF self-assembly at the interface, which in turn can affect the properties of the materials with high surface areas like electrospun matts and scaffolds.

## 1. Introduction

The application of natural biopolymers for the production of new materials with desirable properties is an important task in polymer science. One of the most challenging problems is the creation of new tissue implants [1,2,3]. The biomechanical and chemical properties of such materials should allow the adhesion, growth, and survival of the cells [4,5]. In order to mimic the structure of a natural extracellular matrix, the scaffolds for tissue engineering should have three-dimensional porous architectures. Phase separation [6,7], self-assembly [8,9,10], and electrospinning [1,11,12] are widely used to obtain biomaterials of the required morphology. Electrospinning is an effective method of producing fibers with diameters ranging from a few nanometers to a few micrometers. Natural polymers [12,13,14], proteins [12,15], and synthetic polymers [13,16,17] were transformed by electrospinning into porous scaffolds.

Among these materials, the use of regenerated silk fibroin (SF) from Bombyx mori silkworm cocoons is one of the most popular ways [11,18,19,20,21]. The high biodegradability, biocompatibility, ability to self-assemble, and possibility to obtain various structures, ranging from thin films and fibers to hydrogels, make SF one of the most widely studied biomaterial for tissue engineering. The SF molecule consists of heavy (391 kDa) and light (25 kDa) chains interconnected by a disulfide bond [22]. The heavy chain is composed of repetitive hydrophobic domains with glycine, alanine, and serine amino acid residues [23,24]. The assembly of these domains into β-sheet crystallinity regions contribute to the unique mechanical properties of silk [21,25,26,27,28]. The amorphous hydrophilic part of the secondary structure of the chain ensures the silk elasticity. The number, size, and distribution of crystalline and non-crystalline domains are the factors determining the formation and properties of SF based materials [23,28,29]. Different strategies can be used to control these factors. The mechanical properties of the SF based materials can be enhanced by changing the concentration, temperature, and pH, by the addition of methanol or salt, chemical cross-linking, or the use of guest polymers [21,25,30,31]. Thus, the highly aligned SF/PEO fibers exhibited a 22.0-fold increase in ultimate tensile strength and a 49.3-fold increase in the Young modulus as compared with the randomly oriented SF fibers [21].

Moreover, the use of polymer additives facilitates the electrospinning of silk fibroin-based scaffolds [25,32,33,34,35,36,37,38,39]. SF nanofibers can be obtained without any additives, but then toxic solvents like hexafluoroisopropanol and formic acid are usually used [11]. The materials prepared from pure protein aqueous solutions are more suitable for biomedical applications but require high concentrations of the protein [33,36,40]. The use of polymer additives like PEO and PVP, which can be easily removed from electrospun non-woven matts or films, allows, on the one hand, the SF concentration to be decreased significantly, which is required for stable spinning processes, from 40 to 25 to 5–7 wt%, and, on the other hand, the morphology of the obtained material to be controlled [34]. It was shown that the use of PEO as an additive promotes SF porosity [25,37,41,42], enhancing its biocompatible properties such as protein permeability, enzymatic degradation, and cell migration [32,35,37,41]. The size distribution of surface pores enlarges with the increase in the PEO concentration. SF/PEO blends demonstrate a phase separation, when SF-rich domains are dispersed in a PEO-rich matrix [25,37,42,43]. The SF conformation in domains corresponds mainly to a random coil and/or helical type, whereas the conformation in the matrix part of the surface layer corresponds to a significant extent to β-sheets.

The increase in β-sheet structures in SF occurs in the course of the protein adsorption at the air–water interface [44,45]. The nanoscale engineering of biomimetic thin films and fibers, due to their high surface area, requires information on the surface properties of both the obtained materials and the working solutions [46,47]. The surface tension of the working solutions is usually considered as one of the crucially important factors determining the possibility of the electrospinning process [20,48], while the surface rheological properties strongly influence the stability of SF-based emulsions. SF adsorbs at the air–water and oil–water interfaces and significantly reduces the surface and interfacial tension [46,49,50,51,52,53,54,55,56,57]. The SF adsorption can also increase the amount of β-sheet crystallites [44,45,58]. The formation of β-sheet structures can result in the formation of 2D elastic gels at the interface due to a step-by-step SF self-assembly. In this case, the dynamic surface elasticity is much higher than in dispersions of solid nanoparticles [45,52,54,55,59]. These peculiar surface properties can be attributed to the rearrangement of Silk I to helical Silk III, which is specific to SF at the air–water interface, or to laminated Silk II [44,58].

SF behavior at the interface is influenced by many factors such as pH, ionic strength, and addition of surfactants [52,53,60]. Depending on the concentration and the additive properties, the SF self-assembly can be promoted or depressed. Since the addition of polymers is frequently used to enhance the production of nanostructured SF-based materials, the determination of the surface properties of the mixed solutions containing amphiphilic polymers has great significance. Most studies of SF/polymer mixed systems focus on the morphology and mechanical properties of the obtained materials and only few of them consider the surface properties of SF solutions [54,61]. A probable explanation of the current situation consists of a limited set of experimental methods providing new information on the formation and structure of the adsorption layers of complex liquids. In this study, the impact of two polymers, PEO and PVP, on the dynamic surface properties of SF solutions is considered. The complex approach based on the simultaneous use of the dilatational surface rheology, ellipsometry, and atomic force microscopy allows a determination to be made of the main steps of the adsorption layer formation and the changes in the surface structure under the influence of different concentrations of the added polymers. Although this approach has already been shown to be effective in studying the surface properties of complex fluids, including pure SF solutions [53], this is, to the best of our knowledge, the first time it has been applied to demonstrate how polymers can guide the SF self-assembly process and affect the general relationship between the structure and mechanical properties of complex surface films.

## 2. Materials and Methods

### 2.1. Materials and Preparation

Silk fibroin was isolated from fresh domestic Bombyx mori cocoon shells supplied by a Russian farm cooperative. Sericin was removed by boiling the cocoon shells in 0.2 wt% Na_2_CO_3_ for 30 min. SF stock solutions were prepared according to the procedure outlined in [62]. SF was degummed first by boiling in 0.2% Na_2_CO_3_ (30 min), then dissolved in a 9.3 M LiBr solution and purified by dialysis and centrifugation. The final solution concentration (10 wt%) was obtained gravimetrically.

The investigated solutions in phosphate buffer at pH 7 with an ionic strength of 0.02 M were prepared by dilution of the stock solution. Components of the buffer, NaH_2_PO_4_ and Na_2_HPO_4_ (Sigma Aldrich, Darmstadt, Germany), were used as received.

Two protein concentrations (0.02 mg/mL and 0.2 mg/mL) were chosen because they are characterized by different types of SF supramolecular structures in the surface layer and allow an evaluation of the effect of added polymers on SF aggregation in the surface layer.

For pure SF at the lower concentration of 0.02 mg/mL, the formation of a highly elastic surface layer, characterized with the coexistence of an almost two-dimensional network of thin fibers and more thick ribbons, is observed [53]. At the higher concentration of 0.2 mg/mL, a continuous thick layer consisting of numerous tightly packed fibers is formed. One can expect that further increase in concentration up to the values usually used for obtaining materials will lead to an increase in the formation rate of such structure without significant change in the morphology. The mixed SF–polymer solutions were obtained by adding the required amounts of polyethylene oxide (PEO, Sigma Aldrich, Germany, Mw = 100,000 Da) or polyvinylpyrrolidone (PVP, Sigma Aldrich, Germany, Mw = 40,000 Da) stock solutions to the protein solution. The full procedure can be described as follows. First, the required amount of stock SF solution (10 wt%) was diluted with a buffer solution to obtain a concentration two times higher than the concentration required for measurements (0.02 or 0.2 mg/mL); next, the equal volume of a polymer solution in a buffer was slowly added to the protein solution. The concentration of polymer solution for the preparation procedure was taken to be two times higher than the required one. The final concentration of a polymer in solution under study varied from 0.002 mg/mL to 0.5 mg/mL. To avoid SF aggregation, no vigorous stirring was applied.

PEO and PVP (Sigma Aldrich, Germany) were used without further purification. Triply distilled water was used to prepare all the solutions. The surface tension of the buffer solution was 72.7 mN/m at 21 °C.

### 2.2. Methods

The dynamic surface elasticity was determined by the oscillating ring method, the detailed description of which was given elsewhere [63]. The solution under study wetted the internal surface of a glass ring immersed into the liquid. The motion of the ring up and down along its main axis produced periodical sinusoidal changes in the liquid meniscus and thereby of the liquid surface area inside the ring. The induced surface tension oscillations were measured by the Wilhelmy plate positioned in the center of the ring, and the modulus of the complex dynamic surface elasticity was determined as follows:

(1) $$│E│=\frac{∆\gamma }{∆A/A}$$

where ∆γ is the amplitude of the surface tension oscillations (mN/m) and ∆A/A is the relative amplitude of the surface area change.

The relative amplitude of the surface area of 5% and the oscillation frequency of 0.05 Hz were kept constant during all measurements.

The compression isotherms were measured at a constant rate of the barrier motion of 10 mm/min in a Langmuir trough (KSV NIMA, Espoo, Finland).

In order to evaluate the response of adsorption layers on different deformation amplitudes the Lissajous plots of the dynamic surface tension were obtained by the oscillating bubble method using the instrument PAT1 (Sinterface Technologies, Berlin, Germany). The bubble was formed at the end of a capillary and an automatic dosing system varied the bubble volume and its surface area according to a harmonic law. The amplitude of the surface area oscillations could change from 5 to 20% at a constant frequency of 0.1 Hz. The corresponding oscillations of the surface tension were determined from the oscillations of the drop shape. The data are presented as the dependency of the surface pressure versus relative amplitude of the surface area changes. π was calculated according to the equation π = γ − γ_0_, where γ_0_ is the surface tension of the pure buffer solution and γ is the surface tension of the SF–polymer mixed solution under study.

The ellipsometric angles ψ and Δ were measured by the null-ellipsometer Multiskop (Optrel GBR, Kleinmachnow, Germany) at a single wavelength of 632.8 nm. The angle of incidence of the light was 49°. The reflection of the elliptically polarized light from the liquid surface induces changes in the light polarization and the angles ψ and Δ, which are connected in a model of a homogeneous layer between two homogeneous bulk phases, with the layer refractive index *n*_1_ and a thickness of δ (nm), by the following equation [64,65]:

(2) $$\tan\psi e^{i\Delta }=\tan\psi_{0}e^{i\Delta_{0}}\times \left[1+i\left(\frac{4\pi \delta \cos\varphi_{0}\sin\varphi_{0}n_{2}^{2}M}{\lambda \left(n_{2}^{2}-n_{0}^{2}\right)\left(n_{0}^{2}\sin^{2}\varphi_{0}-n_{2}^{2}\cos^{2}\varphi_{0}\right)}\right)\right]$$

where λ is the wavelength of light (nm), $\psi_{0}$ and $\Delta_{0}$ are the ellipsometric angles (degrees) when the layer thickness is zero, $n_{0}$ and $n_{2}$ are the refractive indexes of the two bulk phases, respectively, $\varphi_{0}$ is the angle of incidence, and $M=n_{0}^{2}+n_{2}^{2}-n_{1}^{2}-n_{0}^{2}n_{2}^{2}/n_{1}^{2}$.

The refractive index and thickness of the surface layer were calculated from experimentally obtained ellipsometric angles by application of a numerical iterative procedure to Equation (2) [66].

The micromorphology of the SF-PEO and SF-PVP adsorption layers was examined using an atomic force microscope (AFM) (NT-MDT Spectrum Instruments, Zelenograd, Russia), operating in a semi-contact mode. The samples were transferred from the air–water interface onto a mica substrate using the Langmuir-Schaefer technique after 12 h of film formation. The samples were then left to dry at room temperature in a desiccator for several days.

## 3. Results

### 3.1. Dynamic Surface Elasticity and Dynamic Surface Tension

**Figure 1:**
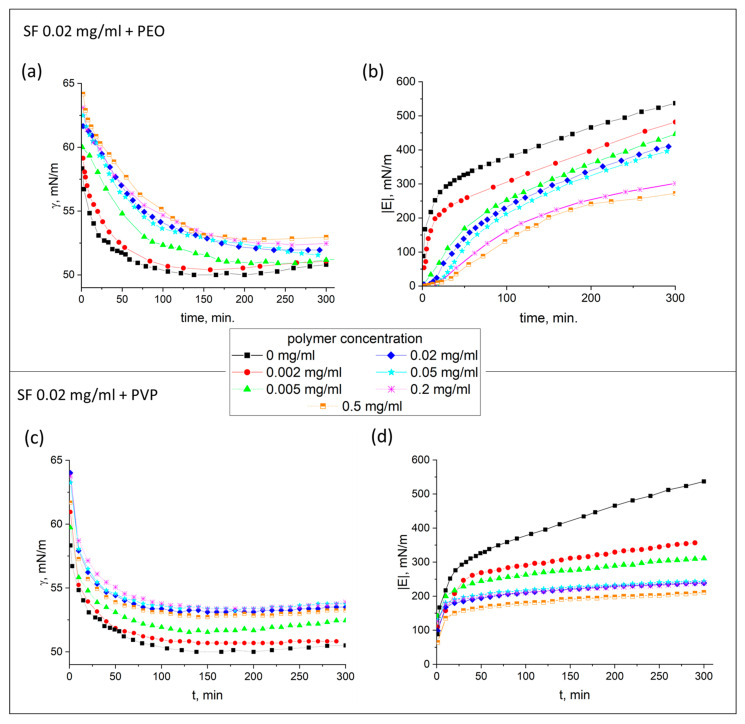
Kinetic dependencies of the dynamic surface tension (**a**,**c**) and the modulus of the dynamic surface elasticity (**b**,**d**) of 0.02 mg/mL SF solutions with addition of PEO (**a**,**b**) and PVP (**c**,**d**), respectively, at polymer concentrations of 0 mg/mL (black squares), 0.002 (red circles), 0.005 (green triangles), 0.02 (blue diamonds), 0.05 (cyan stars), 0.2 (pink snowflakes), and 0.5 mg/mL (orange squares). The standard deviation of the measured parameters was lower than 5%.

**Figure 2:**
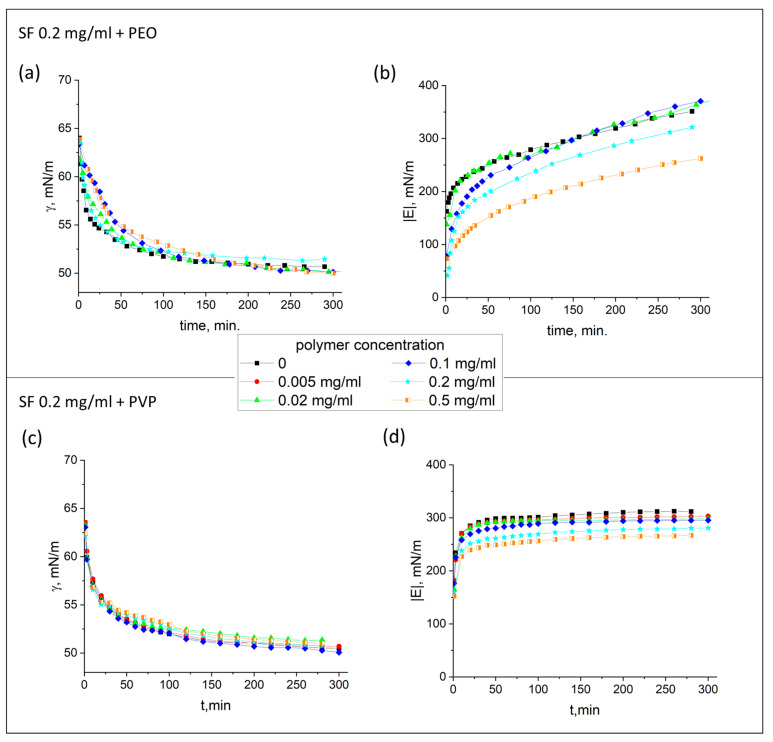
Kinetic dependencies of the dynamic surface tension (**a**,**c**) and the modulus of the dynamic surface elasticity (**b**,**d**) of 0.2 mg/mL SF solutions with addition of PEO (**a**,**b**) and PVP (**c**,**d**), respectively, at polymer concentrations of 0 (black squares), 0.005 (red circles), 0.02 (green triangles), 0.1 (blue diamonds), 0.2 (cyan stars), and 0.5 mg/mL (orange squares). The standard deviation of the measured parameters was lower than 5%.

The dynamic surface tension and dynamic surface elasticity of SF solutions were measured as a function of surface age and polymer concentration at two fixed protein concentrations (Figure 1 and Figure 2). PEO and PVP influence the dynamic surface properties in a similar way. Both polymers decrease the dynamic surface elasticity (Figure 1b,d and Figure 2b,d) and the surface pressure at the lower protein concentration (Figure 1a,c). The dynamic surface tension near equilibrium is approximately the same for both pure protein solutions and is about 50 mN/m. The addition of PEO and PVP to 0.02 mg/mL SF solution leads to a gradual increase in the surface tension up to 53 and 55 mN/m, respectively, for the highest added polymer concentrations. For the higher SF concentration, all the kinetic dependences of the surface tension for pure and mixed solutions are almost identical (Figure 2a,c).

At the same time, the dynamic surface elasticity is more sensitive to the polymer additions for both SF concentrations. The surface elasticity for the lower SF concentration of 0.02 mg/mL decreases to about 40% of the value for pure protein solutions. This distinction is less for the higher SF concentration.

PEO and PVP possess their own surface activity. They can decrease the surface tension of water to 55 and 60 mN/m, respectively. At the same time, the corresponding dynamic surface elasticities are low and do not exceed 5 mN/m [67,68]. At the same time, it can be noted that in all cases, the dynamic surface elasticity exceeds 200 mN/m. Such values are a few times higher than the vales for globular proteins [69] and bring the behavior of the system closer to that of the hard nanoparticles [59]. For pure SF solutions, the dependence of the dynamic surface elasticity has a nonmonotonic character and the maximum values are observed at concentrations of about 0.01–0.02 mg/mL [54,55]. Therefore, the dynamic surface elasticity values for SF–polymer mixtures at a protein concentration of 0.02 mg/mL are usually higher than the corresponding ones for SF–polymer mixtures at a higher protein concentration (0.2 mg/mL). As was shown previously [53], different types of supramolecular structures give different response on deformation. The highest elasticity corresponds to the coexistence of a network of threadlike aggregates and some bigger ribbons. Probably these structures contain regions of the laminated Silk II or helical Silk III of high mechanical strength.

It can be assumed that changes in the dynamic surface tension and dynamic surface elasticity with the polymer concentration are connected with a gradual displacement of protein molecules from the surface layer by the polymer. Moreover, the shift in the ratio between components in the surface layer can lead to the change in supramolecular structure type formed by SF.

### 3.2. Compression Isotherms

**Figure 3:**
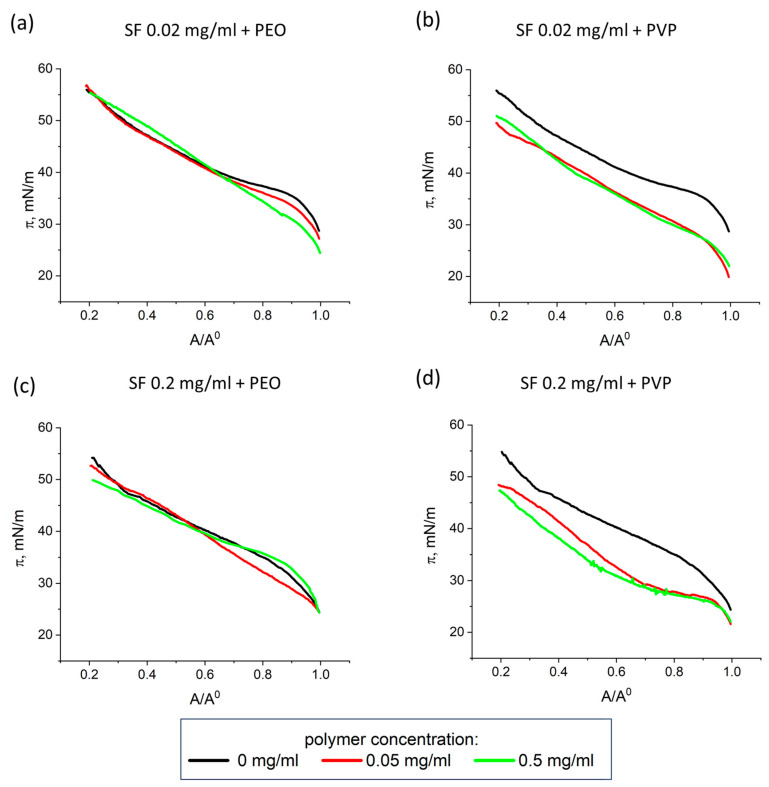
Compression isotherms of SF layers formed from SF solutions with the addition of PEO (**a**,**b**) and PVP (**c**,**d**), respectively. Concentrations of the protein are 0.02 mg/mL (**a**,**c**) and 0.2 mg/mL (**b**,**d**). Concentrations of the polymers are 0 mg/mL (black lines), 0.05 mg/mL (red lines), and 0.5 mg/mL (green lines). The accuracy of surface pressure measurements is ±0.2 mN/m.

The difference between the systems for the lower (0.02 mg/mL, Figure 3a,b) and higher (0.2 mg/mL, Figure 3c,d) SF concentration, respectively, can also be observed for the compression isotherms of the adsorption layers. At the low SF concentration, the compression isotherms almost coincide, regardless of the concentration of PEO. The first points of the isotherms are a little different, since the addition of the polymer leads to a slight increase in the surface tension close to equilibrium. A decrease in the surface area less than 10% results in an abrupt increase in surface pressure up to 35 mN/m and the changes become smoother after that. The difference between these two parts of the compression isotherms decreases at higher PEO concentrations. Similar behavior is observed if PVP is added to the 0.02 mg/mL SF solution, but the isotherms for the mixed solutions are shifted to lower surface pressures.

It is likely that the interaction of SF with PVP is stronger, and the protein cannot be displaced from the surface layer during compression. If, for the 0.02 mg/mL SF concentration, we observe changes from an s-shaped type of curve to almost linear ones, for the 0.2 mg/mL SF concentration, it changes in an opposite direction—from almost linear to s-shaped. All the dependences of surface pressure on relative surface area start from the surface pressure of 28.8 mN/m. For mixed solutions, the increase in surface pressure leads to the same final values as it was previously obtained for a 0.02 mg/mL SF solution (53 mN/m), whereas for pure solutions, it was only about 35 mN/m. It can be assumed that the addition of polymers decreases the concentration of SF in the surface layer and the compression isotherms become similar to the dependences observed for smaller SF concentrations.

According to this assumption, the highest adsorption values are reached for the pure SF solution with a concentration of 0.2 mg/mL, where uniform, thick film consisting of numerous tightly packed fibers is formed. The compression of such a layer can only lead to some local changes in the layer thickness and its structure, leading to very slight surface pressure changes. When PEO or PVP is added to SF, the shape of compression isotherms approaches that of the isotherms for SF solutions with a protein concentration of 0.02 mg/mL. Such compression isotherms can be divided into two parts, attributed to different structures of the protein adsorption layer, where first part corresponds to an increase in concentration of SF supramolecular aggregates. At surface pressures higher than about 35 mN/m, these aggregates start to interact, leading to a further, smoother increase in the surface pressure.

### 3.3. Lissajous Plots

**Figure 4:**
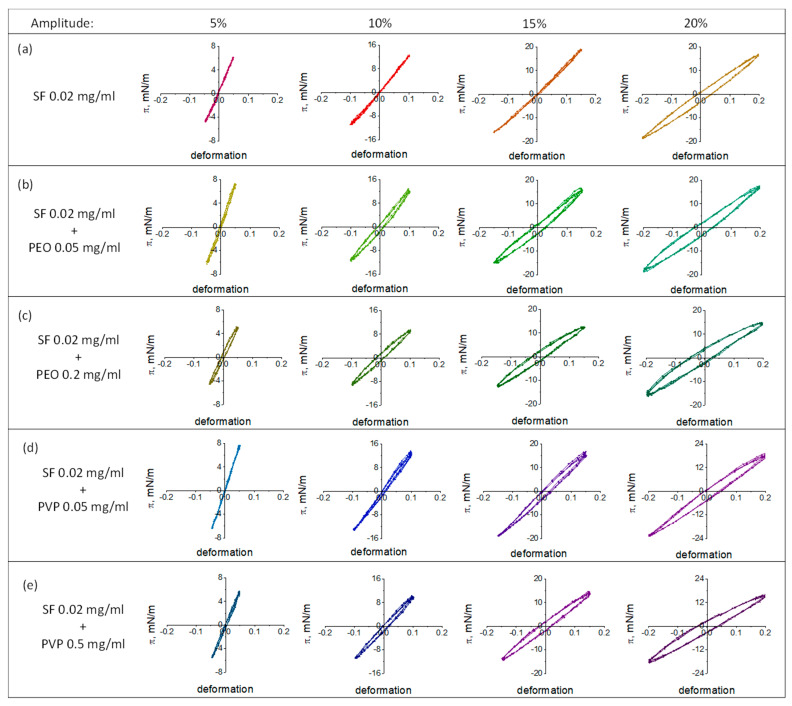
Lissajous plots of surface pressure versus deformation obtained during amplitude sweeps of pure SF adsorption layers (**a**), mixed SF-PEO (**b**,**c**), and mixed SF-PVP (**d**,**e**) adsorption layers at constant protein concentration of 0.02 mg/mL. The accuracy of surface pressure measurements is ±0.5 mN/m.

**Figure 5:**
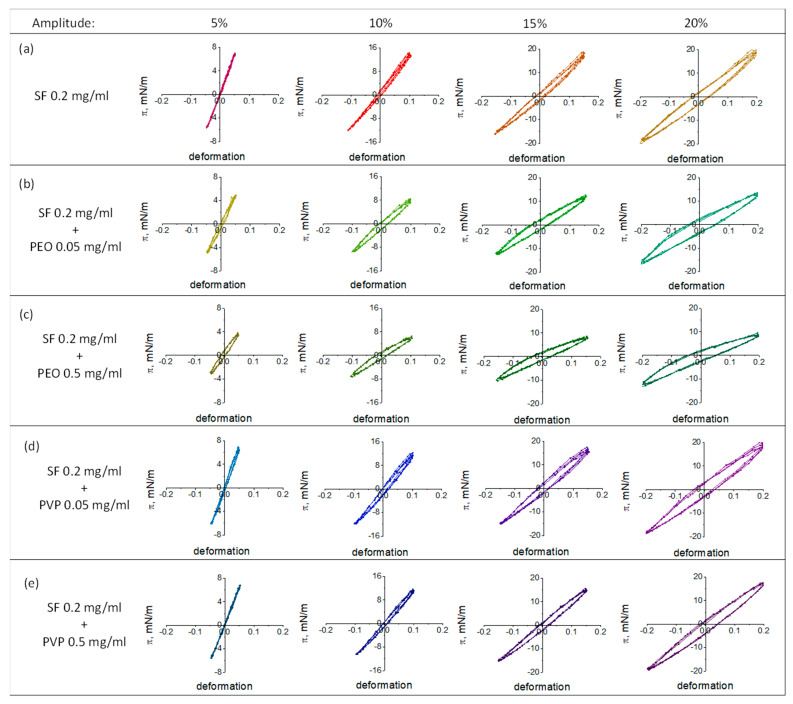
Lissajous plots of surface pressure versus deformation obtained during amplitude sweeps of pure SF adsorption layers (**a**), mixed SF-PEO (**b**,**c**), and mixed SF-PVP (**d,e**) adsorption layers at constant protein concentration of 0.2 mg/mL. The accuracy of surface pressure measurements is ±0.5 mN/m.

The surface pressure can be represented as a function of the surface area during compression and expansion at different deformation amplitudes to obtain Lissajous plots. Figure 4 and Figure 5 show typical Lissajous plots of SF-PEO and SF-PVP adsorption layers at protein concentrations of 0.02 and 0.2 mg/mL, respectively. The shape of the plots strongly depends on the solution composition and the oscillation amplitude demonstrating a purely elastic response, a linear viscoelastic response or a nonlinear asymmetric response at various conditions.

The obtained Lissajous plots for pure SF layers demonstrate highly elastic behavior with almost linear graphs at amplitudes less than 10%. When higher amplitudes (20% for SF concentration of 0.02 mg/mL and >15% for SF concentration of 0.2 mg/mL) are applied, the linear graphs are transformed into ellipses, indicating an increase in the viscous contribution to the dynamic surface elasticity. The addition of polymers leads to a widening of the Lissajous plots at lower deformations. The effect is more pronounced for systems with PEO as compared to those with PVP. It can be assumed that the polymers penetrate the adsorption layer and partly disrupt the elastic film, as formed by pure SF. As a result, a mixed viscoelastic adsorption layer with a looser structure is formed at the interface.

For protein and polymer concentrations of 0.02 mg/mL and 0.5 mg/mL, respectively, the Lissajous plots become less symmetrical and the deviations from a linear graph increase with the amplitude. The response of the surface layer at expansion is different from that at compression. The surface pressures are higher during compression as compared to those at expansion. One can observe a strain hardening at compression and a softening at expansion. The wider plots at higher polymer concentrations indicate a more viscous response due to structural transitions in the surface layer.

The increase in the deformation amplitude and PEO concentration for mixtures with high SF concentrations results in an increase in the slope of the Lissajous plots. The strain hardening of the layer can be connected with the appearance of β-sheet crystallinities of SF in the layer structure increased deformation amplitudes [31,70].

### 3.4. Ellipsometry

**Figure 6:**
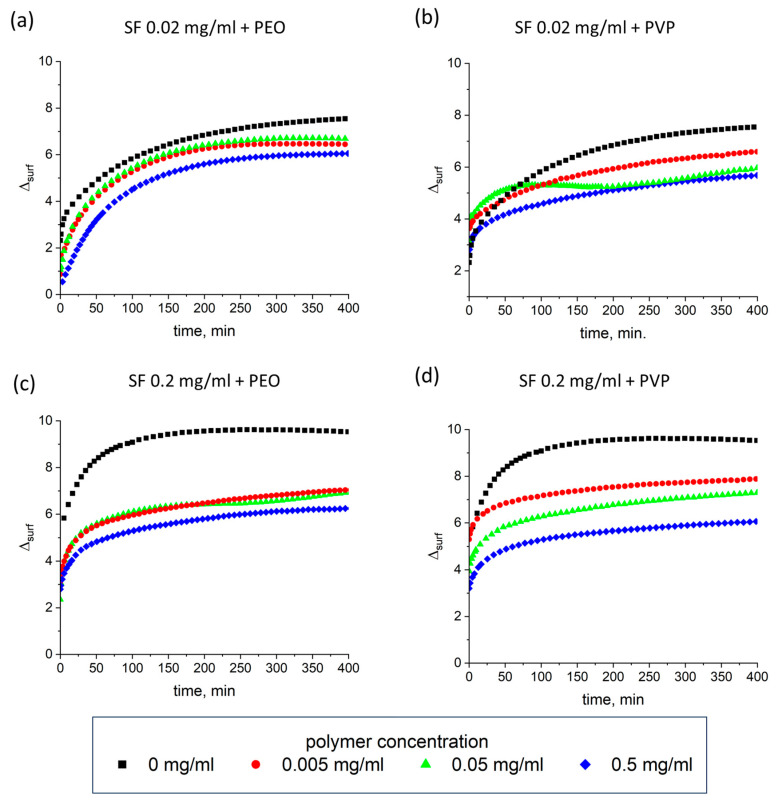
Kinetic dependencies of ellipsometric angle Δ_surf_^0^ for mixed SF-PEO (**a**,**c**) and mixed SF-PVP (**b**,**d**) solutions with polymer concentrations 0 mg/mL (black squares), 0.005 mg/mL (red circles), 0.05 mg/mL (green triangles), and 0.5 mg/mL (blue diamonds). Protein concentration was fixed at 0.02 mg/mL (**a**,**b**) and 0.2 mg/mL (**c**,**d**), respectively. The standard deviation of the measured parameters was lower than 10%.

The addition of PEO and PVP to SF solutions leads to rather close dependencies of the ellipsometric angles (Figure 6). The obtained results show that SF influences stronger the ellipsometric angle Δ than the added PVP and PEO, respectively, corroborating the assumption that the polymers displace SF from the surface layer to a significant extent. The difference between the values of Δ near equilibrium for pure protein solutions and for the mixtures with the highest polymer concentrations is close to 3°, thereby reflecting a significant decrease in the SF surface concentration (Table 1).

The thickness of the layer also decreases with the addition of the polymers, but the changes are less dramatic as it could be expected from the drop of the angle Δ. For pure protein solutions with concentrations 0.02 and 0.2 mg/mL, the thicknesses of the layer near the equilibrium are about 25 and 40 nm, respectively. The addition of PEO in the former case leads to a moderate decrease in the thickness down to approximately 15 nm for the highest polymer concentrations. The influence of 0.5 mg/mL PVP is stronger, leading to a thickness of about 8 nm. The same tendency can be noted for solutions with the higher protein concentration of 0.2 mg/mL. The layer thickness decreases from 40 to 18 and 12 nm for PEO and PVP, respectively. The moderate changes in the layer thickness, especially for low polymer concentrations, allow us to assume that SF molecules are less tightly packed in the surface layer in that case. The monotonic increase in the ellipsometric signal without noticeable fluctuations indicates that a macroscopically uniform structure of the SF layer is preserved in solutions with the added polymers.

### 3.5. Atomic Force Microscopy

**Figure 7:**
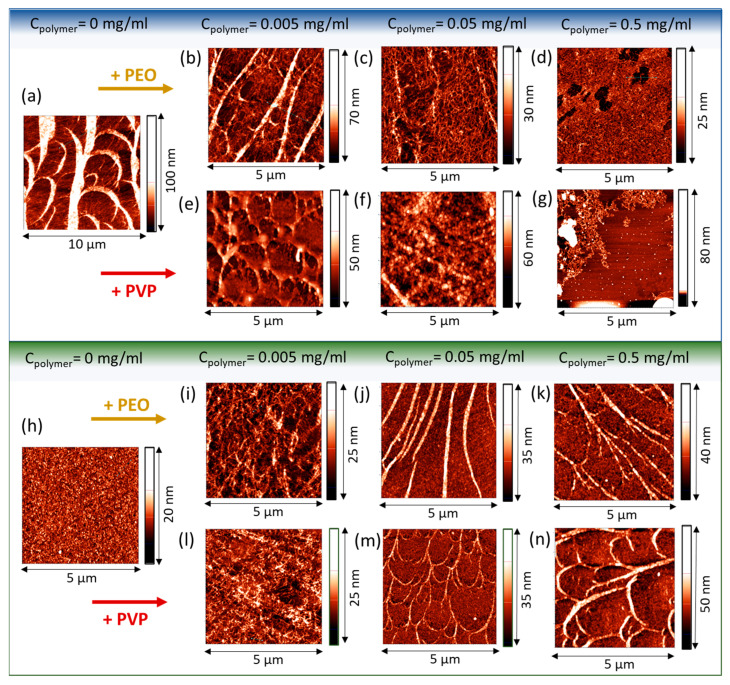
AFM images of SF-PEO and SF-PVP adsorption layers transferred from the air–water interface onto a mica surface. The samples were obtained from solutions with SF concentrations of 0.02 mg/mL (**a**–**g**) and 0.2 mg/mL (**h**–**n**) and polymer concentrations of 0.005 mg/mL (**b**,**e**,**i**,**l**), 0.05 mg/mL (**c**,**f**,**j**,**m**), and 0.5 mg/mL (**d**,**g**,**k**,**n**). The results show different types of structures: tree-like thick ribbons (**a**,**b**,**n**,**j**,**k**,**m**,**n**) and an almost two-dimensional network of thin fibers (**a**–**n**). The width of a ribbon is about 100 (**a**), 30–50 nm (**b**,**j**,**n**), and 20–30 nm (**k**,**m**). The mesh size of the two-dimensional network decreases from less than 10 (**a**) to 20–30 nm (**b**,**c**,**f**,**i**,**l**).

The AFM images demonstrate the changes in the adsorption layer structure upon the addition of the polymers (Figure 7). The results for the lower SF concentration (0.02 mg/mL) are characterized by two types of structures—tree-like thick ribbons and an almost two-dimensional network of thin fibers (Figure 7a). It can be assumed that the tree-like ribbons serve as nuclei of a more homogeneous layer, which is observed for the layers for the higher 0.2 mg/mL solution.

In this case, the layer becomes thicker, as shown by ellipsometry, and presumably consists of branched and tightly packed fibers (Figure 7h).

The additions of both polymers rearrange the observed surface structures. In this case, the morphology resembles that of pure SF layers at lower concentration (Figure 7a) but the number of branches and the size of thick ribbons decreases (Figure 7b). The cross-section of such a ribbon shows that its width decreases from about 100 to 30–50 nm after the addition of 0.005 mg/mL of PEO. The packing of thin threads becomes less tight. The mesh size decreases from less than 10 to 20–30 nm. This is especially noticeable with a further increase in the polymer concentration to 0.05 mg/mL, when the ribbons disappear and one can see a network of thin fibers (Figure 7c). At higher polymer concentrations, some holes with a diameter of about 100 nm appear in the layers in the case of mixed SF-PEO solutions with PEO concentration of 0.5 mg/mL (Figure 7d). The influence of PVP turns out to be stronger and patches of a uniform SF layer are not observed (Figure 7g).

At the higher SF concentration of 0.2 mg/mL, the ribbons in the layer do not disappear at high polymer concentrations (Figure 7i–n). Similarly to the previous case, small PEO and PVP concentrations promote the formation of less dense layers (Figure 7i,l). The further addition of the polymers leads to the formation of structures resembling those for pure SF solutions at the concentration of 0.02 mg/mL with two types of aggregates. Therefore, the main effect of polymers is the reduction in the SF surface concentration with the corresponding changes in the surface morphology. At the same time, the change in the fiber packing density in the regions without ribbons indicates the presence of the polymer molecules in the surface layer.

## 4. Discussion

The complexes of charged polymers with proteins can be used for protein separation, immobilization, or controlled release [71,72,73]. Strong electrostatic and hydrophobic interactions between the components lead to the formation of soluble complexes, aggregates, or coacervates, whose structure and composition can depend on external conditions [74,75].

In contrast, the interactions of proteins with highly hydrophilic nonionic polymers, like PEO and PVP, can be relatively weak. The preliminary adsorption of PEO or PVP decreases sometimes the protein adsorption [76,77,78]. The adsorption from mixed solutions of proteins with PEO and PVP can be described sometimes as a fast adsorption of one of the components followed by its slower displacement by another component [79]. The adsorption of PEO and PVP at the air–water interface can be accompanied by non-monotonic changes in the dynamic surface elasticity [67]. This peculiarity can be connected with the formation of loops and tails in the surface layer leading to a new mechanism of the surface stress relaxation. The polymer chains are squeezed out of the proximal region of the surface layer under compression and pulled in under expansion.

When PEO or PVP is added to SF solutions, all the dependences of the dynamic surface elasticity remain monotonic. The exchange of polymer segments between the proximal and distal regions of the surface layer cannot be noted, presumably due to the high elasticity of the SF layers.

The compression isotherms, ellipsometry, and AFM results show that the SF concentration in the surface layer decreases when PEO or PVP is added to SF solutions. For pure SF solutions, the dependency of the dynamic surface elasticity on protein concentration is non-monotonic and a local maximum corresponds to a protein concentration lower than 0.2 mg/mL [54,55]. The morphology of the adsorption layers in mixed solutions with polymer concentrations of 0.05 mg/mL and higher (Figure 7j,k,n,m) is similar to that of the SF layers having highest elasticity. At the same time, a significant decrease in the dynamic surface elasticity indicates the presence of the polymer in the surface layer.

**Figure 8:**
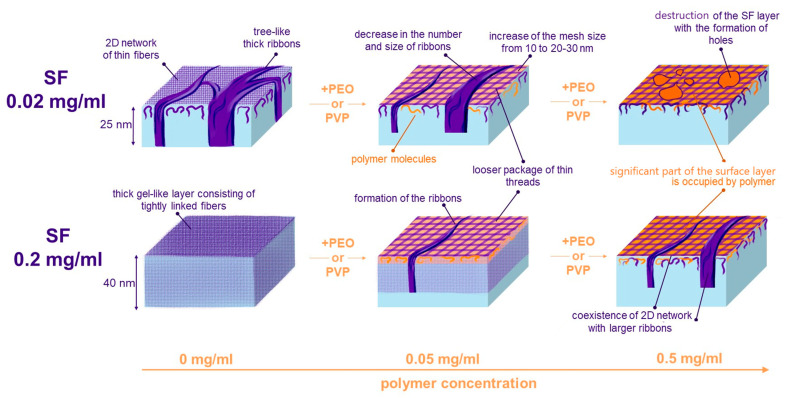
Scheme of the of SF-PEO and SF-PVP adsorption layers formation.

The first step of surface layer formation is determined by the component with the highest concentration (Figure 8). If it is SF, it adsorbs first, and the polymer becomes embedded between the fibers. The polymer loosens the layer structure, and it is possible to observe for the lower SF concentration of 0.02 mg/mL that the denser regions corresponding to tree-like ordered structures gradually disappear (Figure 8).

With increasing PEO and PVP concentrations, the polymer adsorbs faster and can partly displace SF from the surface layer. As a result, a significant area of the surface is occupied by the polymer. For the lower SF concentration of 0.02 mg/mL, this process leads to SF layer destruction. The action of PVP is stronger and the uniform SF film disintegrates, forming separate islands, while for the solutions with PEO at the same concentrations, only some separate layer defects become visible. For the higher SF concentration, the addition of polymer results in a transition from a thick gel-like layer containing numerous tightly linked fibers to a layer characterized by the coexistence of an almost two-dimensional network of numerous branched thin fibers with larger ribbons. In the latter case, the adsorption layer is a combination of the regions with high (ribbons) and low (network) protein concentrations. This finding is in agreement with the previously observed phase separation process in mixed SF-PEO membranes [25,37,42,43]. The addition of PEO induces the formation of SF domains resembling the polymer domains in protein/polymer adsorption layers, similar to the protein/surfactants systems studied in [80].

For pure SF layers, the coexistence of the ribbons and the network of branched fibers corresponds to most elastic layers. It was shown that Silk III modification and highly laminated Silk II regions with a large number of β-sheet crystallites, described in [44,45], give rise to a unique response of the surface layer to dilation, which is similar to that of a layer of solid nanoparticles [59]. The addition of PEO can induce the formation of β-sheets and some changes in the layer morphology, partly explaining the unique mechanical properties of composite SF-PEO materials [25]. Therefore, the polymers like PEO and PVP can be used to regulate the SF self-assembly at the interface, which in turn can affect the properties of the materials with high surface areas like electrospun matts and scaffolds. The resulting increase in the porosity due to the polymer incorporation and the changes in the layer morphology can improve the functional properties of SF materials.

## 5. Conclusions

The simultaneous use of dilatational surface rheology, ellipsometry, and AFM for surface layer analysis allows us to obtain detailed information on PEO and PVP influence on the dynamic surface properties of SF solutions. The polymer determines the type of supramolecular structures at the air–water interface. Depending on the concentrations of SF (0.02 or 0.2 mg/mL) and additives (varying from 0.002 to 0.5 mg/mL), the polymer can increase the mesh size of the fiber network (for all concentrations of additives), induce a transition from a 3D gel-like layer to a two-dimensional network (for SF concentration of 0.02 mg/mL and polymer concentration of 0.05 mg/mL), promote the formation of ribbons in the layer (for SF concentration of 0.2 mg/mL and polymer concentration of 0.05 mg/mL and higher), and even destroy uniform layers (for SF concentration of 0.02 mg/mL and polymer concentration of 0.5 mg/mL). The ellipsometry results and the compression isotherms show that the behavior of the mixed adsorption layers can be similar to a significant extent to that of the SF adsorption layers in solutions of smaller polymer concentrations. At the same time, the strong decrease in the dynamic surface elasticity indicates a significant increase in PEO and PVP concentrations in the surface layer. Since PEO and PVP are typical components of SF based nanomaterials, the results on the surface properties of the protein–polymer mixtures can contribute to the better understanding of the processes of their production and applications.

## Figures and Tables

**Table 1:** Adsorption layer thickness for mixed SF-PEO and SF-PVP solutions. The error corresponds to one standard deviation over 20 thickness measurements at conditions close to the equilibrium.

SF Concentration, mg/mL	PEO Concentration, mg/mL	PVP Concentration, mg/mL	Thickness, nm
0.02	--------	--------	25 ± 0.9
0.02	0.005	--------	20 ± 1.8
0.02	0.05	--------	20 ± 2.0
0.02	0.5	--------	15 ± 2.6
0.02	--------	0.005	18 ± 1.5
0.02	--------	0.05	12 ± 1.2
0.02	--------	0.5	8 ± 0.7
0.2	--------	--------	40 ± 0.6
0.2	0.005	--------	26 ± 1.1
0.2	0.05	--------	20 ± 1.8
0.2	0.2	--------	18 ± 2.1
0.2	0.5	--------	18 ± 2.4
0.2	--------	0.005	26 ± 1.5
0.2	--------	0.05	22 ± 2.0
0.2	---------	0.5	12 ± 1.4

## Data Availability

The data presented in this study are openly available in article.
